# Studies on synthetic LuxR solo hybrids

**DOI:** 10.3389/fcimb.2015.00052

**Published:** 2015-06-18

**Authors:** Daniel Passos da Silva, Hitendra K. Patel, Juan F. González, Giulia Devescovi, Xianfa Meng, Sonia Covaceuszach, Doriano Lamba, Sujatha Subramoni, Vittorio Venturi

**Affiliations:** ^1^Bacteriology and Plant Bacteriology, International Centre for Genetic Engineering and BiotechnologyTrieste, Italy; ^2^Centro de Ciencias da Saude, Instituto de Biofisica Carlos Chagas Filho, Universidade Federal do Rio de JaneiroRio de Janeiro, Brazil; ^3^Istituto di Cristallografia, Unità Organizzativa di Supporto di Basovizza (Trieste), Consiglio Nazionale delle RicercheTrieste, Italy

**Keywords:** LuxR, quorum sensing, LuxR solo, lux box, bacteria, signaling

## Abstract

A sub-group of LuxR family of proteins that plays important roles in quorum sensing, a process of cell-cell communication, is widespread in proteobacteria. These proteins have a typical modular structure consisting of *N*-ter autoinducer binding and *C*-ter helix-turn-helix (HTH) DNA binding domains. The autoinducer binding domain recognizes signaling molecules which are most often *N*-acyl homoserine lactones (AHLs) but could also be other novel and yet unidentified molecules. In this study we carried out a series of specific domain swapping and promoter activation experiments as a first step to engineer synthetic signaling modules, taking advantage of the modularity and the versatile/diverse signal specificities of LuxR proteins. In our experiments the *N*-ter domains from different LuxR homologs were either interchanged or placed in tandem followed by a *C*-ter domain. The rational design of the hybrid proteins was supported by a structure-based homology modeling studies of three members of the LuxR family (i.e., LasR, RhlR, and OryR being chosen for their unique ligand binding specificities) and of selected chimeras. Our results reveal that these LuxR homologs were able to activate promoter elements that were not their usual targets; we also show that hybrid LuxR proteins retained the ability to recognize the signal specific for their *N*- ter autoinducer binding domain. However, the activity of hybrid LuxR proteins containing two AHL binding domains in tandem appears to depend on the organization and nature of the introduced domains. This study represents advances in the understanding of the modularity of LuxR proteins and provides additional possibilities to use hybrid proteins in both basic and applied synthetic biology based research.

## Introduction

Quorum sensing (QS) is a system of bacterial communication which involves the synthesis and detection of chemical molecules called autoinducers to regulate community-specific traits in a cell density dependent manner (Engebrecht et al., 1983; Kaplan and Greenberg, 1987; Fuqua et al., 1994; Fuqua and Greenberg, 2002). Most commonly the canonical QS system in Gram-negative bacteria is composed of genetically coupled *luxI* and *luxR* homologs. The LuxR homolog is the transcriptional regulator that recognizes and binds, at threshold concentration, to the autoinducer molecules [generally *N*-acyl homoserine lactones (AHLs)] synthesized by the LuxI homolog to regulate gene expression (Fuqua et al., 1994; Zhu and Winans, 2001; Fuqua and Greenberg, 2002; Schaefer et al., 2013). LuxR homologs are known to have a modular structure consisting of *N*-ter autoinducer binding domain and *C*-ter helix-turn-helix (HTH) DNA binding domain (Choi and Greenberg, 1991; Hanzelka and Greenberg, 1995). Most of the LuxR homologs require binding to the signal molecule for correct folding/dimerization/stabilization and subsequent activation of target promoter regions (Zhu and Winans, 2001; Vannini et al., 2002; Zhang et al., 2002). Some LuxR proteins are able to fold/dimerize in the absence of the autoinducer to repress the promoter region of target genes but are de-repressed on binding the ligand due to conformational changes (Pearson et al., 1997; Minogue et al., 2002; Medina et al., 2003; Ventre et al., 2003; Steindler et al., 2009). LuxR homologs usually but not always bind to 20-bp palindromic sequence in the promoter regions of target genes centered at the −42.5 position from the transcriptional start site called the *lux* box; it is known that six key nucleotides in the *lux* box are crucial for the complete interaction between the LuxR homologs and the target DNA (Devine et al., 1989; Egland and Greenberg, 1999; Urbanowski et al., 2004; Schuster and Greenberg, 2007; Antunes et al., 2008). Therefore, the two domains of the LuxR protein, the presence of appropriate ligand and important features of the target promoter element are central for LuxR mediated gene regulation.

LuxR homologs without a cognate autoinducer synthase LuxI, namely LuxR solos or orphans, are now known to be common among members of proteobacteria (Chugani et al., 2001; Fuqua, 2006; Subramoni and Venturi, 2009; Subramoni et al., 2011). In some bacteria their presence greatly increases the regulon that is influenced by QS as LuxR solos may bind to endogenous or exogenous AHLs or even novel signals. In other bacteria lacking the canonical QS system, LuxR solos are thought to play a role in interspecies communication (Case et al., 2008; Patankar and Gonzalez, 2009b; Subramoni and Venturi, 2009; Schaefer et al., 2013; Patel et al., 2014). LuxR solos possess the same modular domain organization as the LuxRs of canonical QS systems. Well-studied AHL-binding LuxR solos include QscR of *Pseudomonas aeruginosa* (Chugani et al., 2001; Ledgham et al., 2003; Fuqua, 2006) and SdiA of *Salmonella enterica* and *Escherichia coli* (Ahmer et al., 1998; Michael et al., 2001; Ahmer, 2004). A sub group of LuxR solos are able to respond to yet unknown plant signal molecules (PSM) instead of detecting AHLs. These proteins are found in plant-associated bacteria (PAB) and show changes in one or two highly conserved amino acids of the autoinducer domain which are thought to be important for signal recognition based on structural studies on TraR (Nasser and Reverchon, 2007). These solos were first described in two species from the *Xanthomonas* genus (Ferluga et al., 2007; Zhang et al., 2007). Later additional proteins belonging to this group were described in other bacterial species (Patankar and Gonzalez, 2009a; Subramoni et al., 2011; Chatnaparat et al., 2012) and they are also present in bacteria associated with *Populus deltoides* (Schaefer et al., 2013). *In silico* modeling studies of these LuxR solos based on the crystallographic structures from different LuxR homologs point to the existence of at least three structural patches in the autoinducer binding domain that are involved in signal recognition (Vannini et al., 2002; Zhang et al., 2002; Yao et al., 2006; Bottomley et al., 2007; Chen et al., 2011; Lintz et al., 2011; Covaceuszach et al., 2013; Patel et al., 2014).

In this study we aim to exploit molecular modeling studies for the rational design of synthetic constructs of the LuxR proteins which differ in signal and promoter binding in order to assess the functionality of the LuxR hybrid proteins. We evaluated the ability of a PAB LuxR solo (i.e., OryR) to bind target promoter regions usually activated by canonical LuxR and vice-versa (i.e., canonical LuxR solos activating PAB LuxR solo target promoter region). We also designed synthetic hybrid variants Ola, Lao, and RhLas by exchanging the *N*-ter autoinducer domains of OryR, LasR, and RhlR and evaluated their ability to bind and regulate specific promoter regions in the presence of the new cognate signal. Finally different synthetic LuxR hybrid variants Dahl1 and Dahl2 containing two *N*-ter autoinducer domains and one *C*-ter DNA binding domain were assessed for their capacity to respond to one or two AHLs signals activating target promoters. This study provides new information about the modularity and signal specificity of LuxR proteins and opens new options for the use of LuxR hybrids in engineering bacterial synthetic regulation systems.

## Materials and methods

### Bacterial strains, plasmids, and growth conditions

*Pseudomonas aeruginosa* PUPa3 and *Escherichia coli* strains used in this study are listed in Table S1. *P. aeruginosa* PUPa3 and its derivatives were routinely grown in Luria Bertani (LB) broth and when required antibiotics were added in the following concentrations: 100 μg ml^−1^ nitrofurantoin (Nif), 100 μg ml^−1^ ampicillin (Ap), 300 μg ml^−1^ kanamycin (Km), 100 μg ml^−1^ gentamycin (Gm), 100 μg ml^−1^ tetracycline (Tc) and 250 μg ml^−1^ chloramphenicol (Cm). *E. coli* DH5α and M15 were routinely grown in LB broth and antibiotics were added in the following concentrations when required: 50 μg ml^−1^ Km, 10 μg ml^−1^ Gm, 10 μg ml^−1^ Tc, and 100 μg ml^−1^ Cm. External addition of rice macerate in LB was performed as described by Gonzalez et al. (2013) and AHLs were externally added at a concentration of 1 μM, unless stated otherwise.

### Recombinant DNA techniques, plasmids construction, and gene synthesis

All recombinant DNA techniques were performed as described in Sambrook and Russell (2001), plasmids were purified by using EuroGold columns (EuroClone, Italy) and plasmid constructs were sequenced by Macrogen (Europe). All plasmids and primers used in this study are described in Table S2.

**Figure 1:**
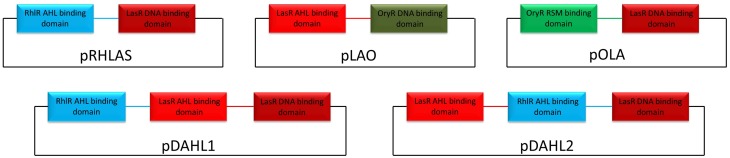
**Schematic representation of the hybrid variants exploited in this study**. DNA sequences belonging to LasR, RhlR, and OryR are colored in red, light-blue, and green respectively. Linker regions between the functional domains are colored accordingly.

Gene synthesis was performed by GeneArt® (Life Technologies, Italy) or by GenScript (GenScript, USA) and all nucleotide sequences are listed in Table S3. Construction of pLAO and pOLA was performed in a sequential subcloning where first the synthesized gene from pMX was cloned in pBBR1MCS2 using HindIII/SacI and then transferred to pBBR1MCS3 using XhoI/SacI. For the construction of pDAHL1 the synthesized gene was transferred from cloning vector pMX to pBBR1MCS2 using HindIII/SacI, resulting in the expression vector pDAHL1. To generate pDAHL2 the synthesized gene was transferred from pUC57 to pBBR1MCS2 using HindIII/SacI, resulting in the creation of pDAHL2. For the generation of pRHLAS amplifications and sequential cuts were performed as follows. First *lasR* and *rhlR* were amplified from *P. aeruginosa* PUPa3 using the pair of primers LasR Fw/LasR Rv and RhlR Fw/RhlR Rv to generate pLASR and pRHLR, respectively. Amplicons were cloned into pGEM® T Easy and sequenced. Constructs were then transferred to pBBR1MCS2 using HindIII/SacI. From this point pLASR was digested using HindIII/BsrBI/SacI generating two fragments which were the AHL-binding domain (approximately 480 bp) and the DNA-binding domain (approximately 230 bp) and the latter eluted and saved. The construct pRHLR was used as template to amplify the AHL-binding domain using the pair of primers T3/RhlRSmaI Rv and the amplicon was cloned in pGEM® T easy. It was then digested with HindIII/SmaI generating a fragment of approximately 520 bp. The AHL-binding domain of RhlR was then cloned HindIII/SacI in frame with the DNA-binding domain of LasR in pBBR1MCS2 generating the construct pRHLAS. All hybrid constructs are schematically represented in Figure 1.

### β-galactosidase and β-glucuronidase assays

β-galactosidase activities were determined during growth in LB as described by Miller (1972) with the modifications of Stachel et al. (1985). All experiments were performed in triplicate. Statistical analysis was performed using Student's *t*-test. The growth curves of all strains were determined and no significant differences were observed (Figure S3). Only the *E. coli* strain harboring pDAHL1 displayed a slightly longer lag phase under the conditions we tested.

The β-glucuronidase activities of overnight cultures of *E. coli* DH5α or *P. aeruginosa* PUPa3 with the pSS122 reporter plasmid carrying different promoters were determined as follows. Bacterial cells were centrifuged and re-suspended in 600 μl of GUS buffer (50 mM sodium phosphate pH 7.0, 1 mM EDTA, 14.3 mM 2-mercaptoethanol). After this 23 μl of 3% Triton X-100 in GUS buffer and 23 μl of 3% sodium lauryl sarcosinate in GUS buffer were added to the samples, the preparations were incubated at 30°C for 10 min, and then 100 μl of 25 mM *p*-nitrophenyl-β-d-glucuronic acid (PNPG) (Sigma) were added. The reaction was stopped by adding 280 μl of 1 M Na_2_CO_3_ after sufficient yellow color had developed. Both the optical densities at 595 nm (OD_595_) of the bacterial cultures and the OD_415_ of the samples, measuring the intensity of the yellow color developed by the β-glucuronidase reaction (OD_415PNPG_), were determined. 1 Miller unit of β-glucuronidase activity was defined as follows: 1 Miller unit = 1000 × {[OD_415PNPG_ − (1.75 × OD_595_)]/(*t* × *v* × OD_595_)}, where *t* is the time of the reaction (in minutes), *v* is the volume of the culture assayed (in milliliters), OD_595_ is the cell density just before the assay, and 1.75 is the correction factor. All measurements were done in triplicate. Statistical analysis was performed using Student's *t*-test.

### Homology modeling

Five web-based servers were exploited to build the 3D homology models and the top-score models generated were then ranked and validated by the protein model quality predictors AIDE (Mereghetti et al., 2008) and ProQ (Wallner and Elofsson, 2003), including PSIPRED (Buchan et al., 2010) for secondary structure prediction.

In details HH-pred (Biegert et al., 2006) produced the top-score model for full length LasR and synthetic hybrids Lao and Ola (being the predicted LGscore and MaxSub values of 5.081, 4.208 and 2.608, 0.459, 0.323, and 0.211, respectively, and the statistical indicators TM-score and RMSD being 0.74, 0.76, and 0.70, and 5.08, 5.68, and 8.42 Å, respectively). The top scored model for full length RlhR was generated by InFold (Roche et al., 2011) (being the predicted LGscore and MaxSub value of 4.445 and 0.503, respectively, and the statistical indicators TM-score and RMSD being 0.71 and 6.13 Å, respectively); while Phyre2 (Kelley and Sternberg, 2009) produced the top scored model for RHLAS (being the predicted LGscore and MaxSub value of 4.624 and 0.495, respectively, and the statistical indicators TM-score and RMSD being 0.67 and 7.01 Å, respectively). All templates that have been used for homology modeling are listed in Table S3.

## Results

### Both AHL and non-AHL binding LuxR proteins regulate foreign promoters

**Figure 2:**
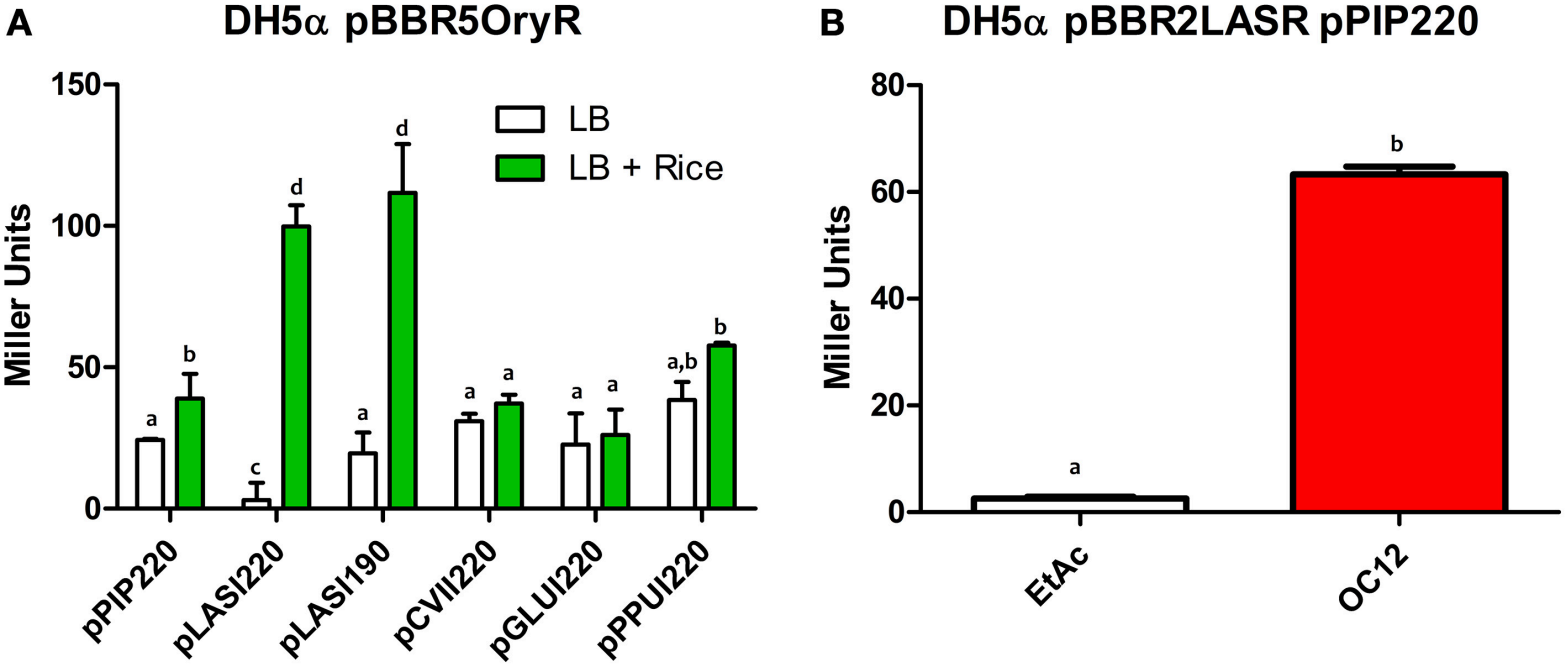
**β-galactosidase assays showing reporter expression levels regulated by** ***pip*****,** ***lasI*****,** ***cviI*****,** ***gluI*****, and** ***ppuI*** **promoters in** ***E. coli***. **(A)** β-galactosidase measurements of promoter activities in the presence of native OryR (pBBR5OryR) of Xoo using plasmids pPIP220, pLASI220, pCVII220, pGLUI220, and pPPUI220, in the absence (EtAc) or presence of total rice macerate at 5% v/v. **(B)** β-galactosidase measurements of *pip* promoter activity in the presence of native LasR (pBBR2LasR) of *P. aeruginosa* strain with pPIP220 in the absence (EtAc) and presence of the cognate signal molecule (OC12-HSL) at a concentration of 1 μM. All experiments were performed on triplicate, the means, and errors are shown and the statistical analysis were calculated using Student's *t*-test (*P* = 0.05). Distinct letters (a–d) indicate statistically different values.

In order to assess whether PAB LuxR solos could bind and activate target promoters of a canonical LuxR protein, β-galactosidase assays were carried out with specific promoter-*lacZ* reporter fusion constructs as described in the Materials and Methods section. All strains harboring the plasmid constructs did not display any significant alteration in growth and in the transition to the stationary phase (Figure S3). In these assays, we tested the ability of the PAB LuxR solo (OryR) of *Xanthomonas oryzae* pv. *oryzae* (Xoo) to activate promoter regions of *lasI* and *rhlI* in *E. coli*. As expected OryR activated reporter expression regulated by the promoter of the Xoo proline iminopeptidase gene (*pip*; pPIP122 or pPIP220) which is known to be its target gene in the presence of rice-plant macerate (Ferluga and Venturi, 2009). Additionally, among the several promoters tested OryR was able to activate the expression of the *lasI* promoter which is known to be regulated by LasR in the presence of OC12-HSL (pLASI220 and pLASI190) (Rampioni et al., 2007; Babic et al., 2010) (Figure 2A). In a reverse experiment, LasR and RhlR were expressed in *E. coli* and tested for their ability to bind and activate the promoter region of Xoo *pip* (pPIP220). Reporter expression was not detected in the presence of any of the proteins tested (Figure S1) except by LasR in the presence of its cognate signal OC12-HSL (Figure 2B).

Overall these data suggest that the operator elements of the *lasI* promoter and of the promoter regulated by OryR are sufficiently similar to enable recognition and activation by either OryR or LasR.

### Domain switching maintains modular functionality of hybrid LuxR proteins

**Figure 3:**
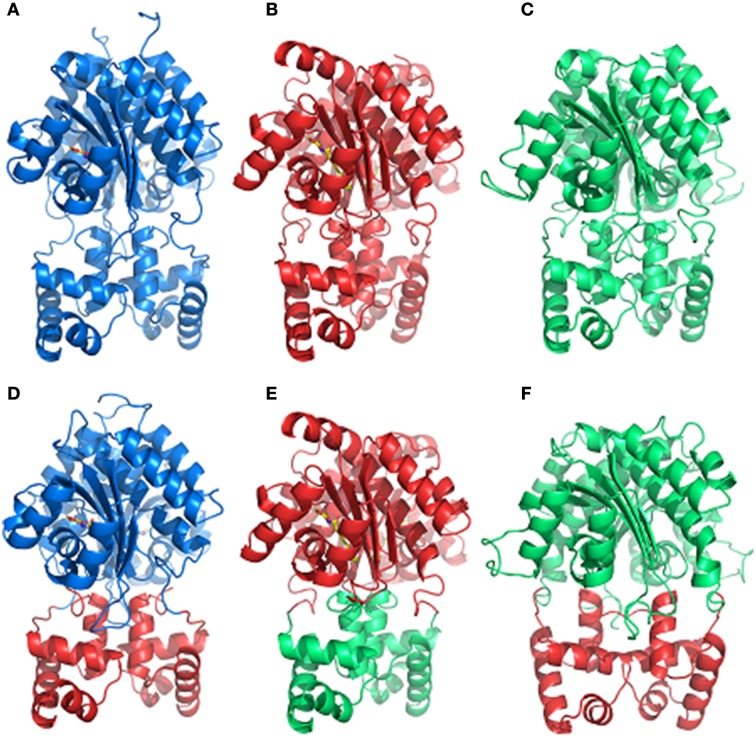
**Comparison of the dimeric assembly of the 3D structure-based homology models of the full-length LuxR homologs and of the synthetic hybrids variants**. **(A)** RhlR, **(B)** LasR, **(C)** OryR, **(D)** Rhlas, **(E)** Lao, **(F)** Ola. The color code is as in Figure 1. The carbon, nitrogen and oxygen atoms of AHLs, are colored in yellow, blue, and red, respectively. Figures produced by Pymol (DeLano, 2002).

We designed synthetic hybrid proteins variants by using domains of native full-length proteins LasR and RhlR from *P. aeruginosa*, and OryR from *X. oryzae* pv. *oryzae* as described in the Materials and Methods section (Figure 1). In order to validate the structural consistency of the synthetic hybrids, the homology models of each native and hybrid protein were constructed and their capability to form a functional dimeric assembly exploited. In detail, the previously obtained OryR model (Covaceuszach et al., 2013) and the homology of full length LasR and RhlR models were compared with those built on the basis of the designed synthetic hybrids, i.e., Rhlas (AHL-binding domain of RhlR and DNA-binding domain of LasR), Lao (AHL-binding domain of LasR and DNA-binding domain of OryR) and Ola (PSM-binding domain of OryR and DNA-binding domain of LasR). As shown in Figure 3, in all the hybrids most of the linker after the last alpha-helix of the autoinducer binding domain (in details 7aa in either LasR and OryR and 9aa in RhlR) were maintained to guarantee the control of the orientation of the respective HTH DNA binding domains, allowing specific promoter recognition in response to the cognate signaling molecule bound by the autoinducer binding domain.

**Figure 4:**
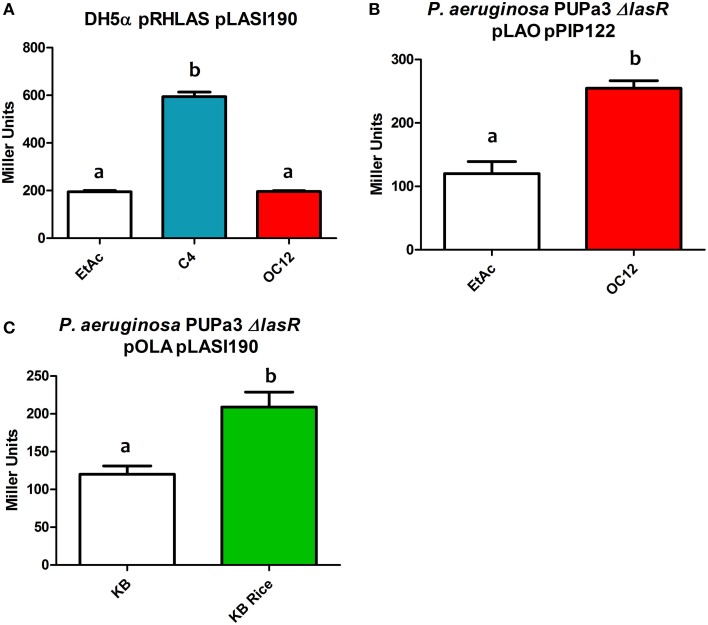
**β-galactosidase assays showing reporter expression levels regulated by** ***lasI*** **and** ***pip*** **promoter in different strains. (A)** β-galactosidase measurement of promoter activity in the presence of the hybrid protein Rhlas (pRHLAS) using the reporter plasmid pLASI190 in the absence (EtAc) or in the presence of AHLs (C4 and OC12-HSL) at a concentration of 1 μM in *E. coli*. **(B)** β-galactosidase measurement of promoter activity in the presence of the hybrid protein Lao (pLAO) using the reporter plasmid pPIP122 in the absence or presence of OC12-HSL at the concentration of 1 μM in *P. aeruginosa* PUPa3Δ*lasR*. **(C)** β-galactosidase measurement of promoter activity in the presence of the hybrid protein Ola (pOLA) with plasmid pLASI190 in the absence or presence of total rice macerate at 5% v/v in *P. aeruginosa* PUPa3Δ*lasR*. All experiments were performed on triplicate, the mean, and error are shown and the statistical analysis were calculated using Student's *t*-test (*P* ≤ 0.05). Distinct letters (a and b) indicate statistically different values.

In order to evaluate the effects of interchanging the AHL-binding domains of LasR and RhlR hybrid construct pRHLAS was tested for its ability to regulate gene expression in response to cognate signals of both LasR and RhlR. The resulting Rhlas activated expression of the *lasI* promoter only in the presence of C4-HSL (Figure 4A). This result shows that by interchanging the AHL domains of QS LuxR homologs the hybrid protein remains functional and retains the ability to respond to the cognate signal recognized by the AHL-binding domain to activate gene expression, in this case the target promoter of LasR.

We were also interested to determine whether the modularity of domains would also be maintained in hybrid proteins that contained autoinducer binding domains with relatively distinct ligand selectivity as for example AHL vs. plant-derived signal molecules. We tested this by interchange of the PSM-binding domain of OryR and the AHL-binding domain of LasR. The hybrid proteins Lao and Ola (Figures 3E,F respectively) were tested for their ability to respond to OC12-HSL and rice macerate, respectively. For both Lao and Ola we observed activation of reporter expression using pPIP122 and pLASI190 (that carry P_*pip*_ and P_*lasI*_, respectively) in the presence of the cognate signals of their *N*-ter domains (Figures 4B,C). These results indicate that although activation levels are not as high as in a hybrid protein of two canonical LuxR proteins that responds to AHLs, Lao, and Ola are still functional.

### Hybrid LuxR proteins with two autoinducer binding domains in tandem are functional

**Figure 5:**
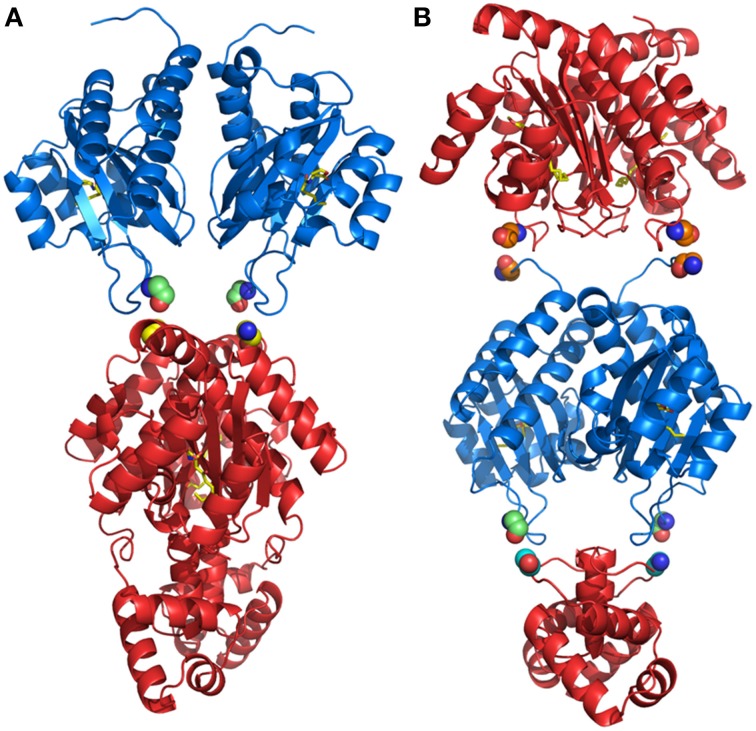
**Comparison of the dimeric assembly of the 3D structure-based homology models of the synthetic hybrids containing two AHL-binding domains. (A)** Dahl1 and **(B)** Dahl2. The color code is as in Figure 1. The corresponding linking flexible regions in the Dahl1 and Dahl2 hybrids variants are colored in green/yellow and in orange and in green/cyan, respectively. The main chain atoms of the residues that are joined together in the synthetic hybrids are shown as spheres. The carbon, nitrogen, and oxygen atoms of AHLs, are colored in yellow, blue, and red, respectively. Figures produced by Pymol (DeLano, 2002).

Results from our experiments with hybrid LuxR proteins indicated that swapping modular domains of these proteins did not lead to loss of function and actually conferred specificity toward the cognate signal of the interchanged *N*-ter autoinducer binding domain. It was also of interest to test the effects of addition of a second autoinducer binding domain of full-length LuxR proteins and to examine their ability to respond to the cognate signals of both autoinducer binding domains by monitoring reporter expression regulated by a target promoter (i.e., P_*lasI*_). The hybrid synthetic LuxR proteins generated as part of this study are represented in Figure 1. Their structural consistency was exploited by homology modeling. In detail, the dimeric assembly of RhlR and LasR homology models showed that the respective positions and distances of the joining aminoacids (whose main chain atoms are highlighted by spheres) were consistent with a likely dimeric assembly of Dahl1 and Dahl2 (depicted in Figures 5A,B, respectively), especially taking into account the flexibility of the regions at the *N*-ter of the proteins and at the level of the linker regions of the autoinducer binding domains that are combined in these hybrids.

**Figure 6:**
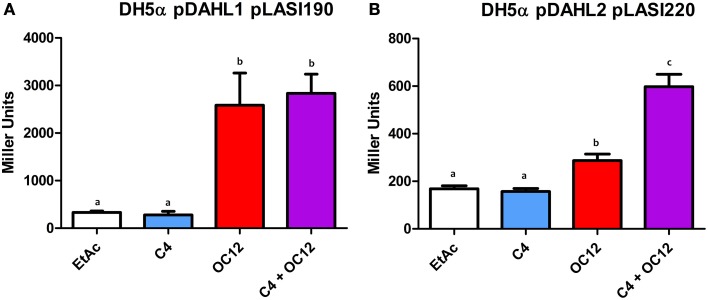
**β-galactosidase assays showing reported expression levels regulated by the** ***lasI*** **promoter in different conditions. (A)** β-galactosidase measurement of the promoter activity in the presence of the hybrid protein Dahl1 (pDAHL1) with reporter plasmid pLASI190 in the absence (EtAc) or in the presence of AHLs (C4 and/or OC12-HSL) at a concentration of 1 μM. **(B)** β-galactosidase measurement of the promoter activity in the presence of the hybrid protein Dahl2 (pDAHL2) with reporter plasmid pLASI220 in the absence or presence of AHLs (C4 and/or OC12-HSL) at the concentration of 1 μM. All experiments were performed on triplicate, the mean, and error are shown and the statistical analysis were calculated using Student's *t*-test (*P* ≤ 0.05). Distinct letters (a and b) indicate statistically different values.

To assess the functionality of Dahl1, in which the *N*-ter AHL-binding domain of RhlR was placed upstream the native LasR, we used C4-HSL alone, OC12-HSL alone or a mixture of both signal molecules. We observed that C4-HSL alone was not able to induce activation of the *lasI* promoter, whereas OC12-HSL induced reporter expression (Figure 6A). The combination of both AHLs neither increased nor decreased the levels of promoter activation, compared to having OC12-HSL alone, indicating that the presence of the newly added RhlR AHL-binding domain did not interfere with the activity of the native LasR protein.

We also constructed a similar double AHL-binding domain protein in which the two autoinducer binding domains were inverted with respect to Dahl1, resulting in the Dahl2 hybrid (Figure 1). Surprisingly by using Dahl2 we were not able to detect activity when the protein was exposed only to C4-HSL. In presence of only OC12-HSL the promoter expression levels were raised around 1.5 times. However, when both signals were added concomitantly we could measure promoter activation levels up to 3 times higher than in the control experiments (Figure 6B). These results suggest that differently from Dahl1, the full activity of this hybrid protein was only possible when both *N*-ter autoinducer binding domains had their cognate signal molecules present.

## Discussion

LuxR homologs are important proteins that are involved in gene regulation in Gram-negative bacteria (Whitehead et al., 2001; Fuqua and Greenberg, 2002). Many studies indicate that these proteins usually possess specificity toward one or few cognate molecules conferring a controlled response to self or non-self signals (Zhu and Winans, 2001; Minogue et al., 2002; Urbanowski et al., 2004; Yao et al., 2006; Zhang et al., 2007; Ferluga and Venturi, 2009; Subramoni et al., 2011). Although much is known about which are the cognate signal molecules and the set of genes being regulated by several LuxR homologs, little is known about the modular activity of these proteins (Choi and Greenberg, 1991; Hanzelka and Greenberg, 1995; Antunes et al., 2008; Ahumedo et al., 2010; Lintz et al., 2011; Covaceuszach et al., 2013). Our results indicate that the LuxR homolog OryR can activate the target promoter of LasR and vice versa. The analysis of the promoter region of *lasI* and *pip* shows that they are very similar, especially because of the conserved CT(N_12_)AG motif present both in the *las* and the *pip* boxes (Figures S2A,B) (Schuster et al., 2004; Schuster and Greenberg, 2007). Our experiments with hybrid synthetic LuxR proteins variants with exchanged domains of LasR and RhlR showed that the proteins were functional and retained specificity for the cognate signal molecule of the *N*-ter autoinducer binding domain. Furthermore, when two *N*-ter autoinducer binding domains were present in tandem, the hybrid proteins retained their functionality. The activity of these double autoinducer binding domain containing hybrid LuxR proteins were however dependent not only on the cognate signal molecule(s) but also on the sequential order of the domains. In fact, synergistic effect due to the presence of both cognate signal molecules was observed for one of the two hybrid proteins, i.e., Dahl2. These observations provide new information regarding the modularity and signal specificity of non-native hybrid LuxR proteins.

The ability of LasR and OryR to regulate foreign promoters in response to their cognate signal molecules suggests that the operator sequences of their promoters are close enough to facilitate promoter activation. Therefore, combinations of either of these promoters and the LuxR homolog proteins with their cognate signal molecules may be exploited for engineering signaling modules or synthetic microbial communities. For example, in the construction of complex regulation systems in synthetic biology involving two or more LuxR homolog proteins that respond to different signal molecules but regulate the same targets. On the other hand cross-promoter specificity of LuxR homolog proteins that respond to AHL and non-AHL signal molecules such as plant derived molecules has important implications for inter-bacterial interactions in a common niche colonized by different bacterial species. Our previous results have demonstrated that 15 different AHLs (including C4-HSL and OC12-HSL) did not result in *pip* promoter activation by OryR and furthermore did not interfere with OryR-activation via the plant signal (Ferluga and Venturi, 2009). In addition biochemical tests have shown that none of the many AHLs tested could solubilize OryR (Ferluga et al., 2007). These results clearly indicate that the two *N*-terminal binding domains do not undergo competition with respect to their ligands making these hybrid proteins specific for a response signal.

LuxR hybrid proteins that had their domains exchanged proved to be still functional when they were presented with the signal molecule known to activate the interchanged *N*-ter autoinducer binding domain. Even swapping the autoinducer binding domain between a protein that responds to plant derived signal molecules (i.e., OryR) and a protein that responds to OC12-HSL (i.e., LasR) conferred the ability to the new hybrid protein to respond to the new cognate signal molecule but kept the specificity toward its native promoter region. The ability to control the molecules, to which the LuxR homolog proteins respond, could allow bacteria to respond not only to its cognate QS signal molecule, but also to an exogenous signal molecule.

The addition of a second ligand binding domain upstream a native LuxR homolog proved to work differently depending on the constructed protein. Dahl1, that is comprised of native LasR with an *N*-ter AHL-binding domain of RhlR added upstream, proved to be functional only when the LasR cognate signal molecule was exogenously provided (i.e., OC12-HSL). On the other hand the construct, in which the AHL-binding domain of RhlR was placed in between the AHL-binding and the HTH-DNA binding domain of LasR (Dahl2), showed full activity only in the presence of both signals. This latter result could pave the way to the construction of hybrid proteins which will respond not to one but to two different AHL signals triggering a second level of control or even leading to a complex regulation system, as for example by adding the PSM-binding domain of OryR in between the two domains of LasR. Our results also shows that by simply adding a second AHL-binding domain in a native LuxR homolog might not always confer a second level of control making clear that more studies are required to elucidate the importance of the *N*- and *C*- ter domains of LuxR homolog for their transcriptional activity. It is tempting to speculate that the outcome of this response is the likely result of the interplay between the protein folding, solubility and stability properties of the autoinducer binding domains in the absence of the cognate AHL that differs in the Dahl1 and Dahl2 hybrid variants. Indeed LasR is highly insoluble in the absence of OC12-AHL, while RhlR is known to be soluble, even if not active, in the absence of C4-AHL. Therefore, in both cases the solubility and the associated functionality of the two hybrids are likely to be strictly dependent on the presence of OC12-AHL, essential for the LasR AHL-binding domain to be soluble. However, in the Dahl1 hybrid the conformational change allowing a proper HTH DNA-binding domain orientation is likely to be only dependent on the LasR AHL-binding domain (due to its proximity to the HTH DNA binding domain) and therefore only on the presence of OC12-AHL. On the contrary in the Dahl2 variant the presence of OC12-AHL that is known to trigger the folding of the LasR AHL-binding domain might be suboptimal in conferring the proper HTH DNA-binding domain orientation due to the presence of the soluble RhlR AHL-binding domain just upstream of the HTH DNA binding domain. The presence instead of both AHLs might guarantee the overall proper folding and stabilization and therefore the optimal transcriptional activity.

In summary, our study evidences that OryR and LasR possess promoter regions with highly similar *lux*-like boxes. It also shows that domains of different LuxR subfamilies which respond to different signal molecules can be exchanged indicating a most probable common ancestor. Lastly the results obtained here using tandem double AHL-binding domains could pave the way to the design of novel proteins which are functional when they respond to more than one type of AHLs. These results open new avenues for future research on the importance of the modularity of LuxR proteins.

### Conflict of interest statement

The authors declare that the research was conducted in the absence of any commercial or financial relationships that could be construed as a potential conflict of interest.
