# Supplementary material for: Studies on synthetic LuxR solo hybrids
Source: Front Cell Infect Microbiol. 2015 Jun 18;5:52. doi: 10.3389/fcimb.2015.00052 (PMC4471428; doi:10.3389/fcimb.2015.00052)
Supplement: Supplementary file 1 [file Table1.DOCX]

**Tables S1_S2_S3.** List of bacterial strains, plasmids, primers and synthetic constreucts used in this study.

| **Strains** | **Relevant characteristics** | **Source** |
| --- | --- | --- |
| ***E. coli* DH5α ^TM^** | Φ80*lacZ*ΔM15, Δ(*lacZYA*-*argF*) U169, *recA*1, *endA*1, *hsdR*17 (rK^–^, mK^+^), *phoA,* *supE*44, λ^–^, *thi*-1, *gyrA*96, *relA*1 | Invitrogen-LifeTechnologies^TM^ |
| ***E. coli* DH5α ^TM^ pRK2013** | Derivative of *E. coli* DH5α^TM^ containing pRK2013 (Km^r^ oriColE1 RK2-Mob^+^ RK2-Tra^+^) | ([Figurski and Helinski, 1979](#_ENREF_4)) |
| ***Pseudomonas aeruginosa* PUPa3** | Amp^r^, Nif^r^ | ([Kumar *et al.*, 2005](#_ENREF_7)) |
| ***P. aeruginosa* Δ*lasR*** | *lasR*::Km^r^ of *P. aeruginosa* PUPa3 | ([Steindler *et al.*, 2009](#_ENREF_10)) |

Ap^r^, Km^r^ and Nif^r^ resistant to ampicillin, kanamycin and nitrofurantoin, respectively.

**Table S2.** List of plasmids and primers used in this study.

| **Plasmids** | | | **Relevant characteristics** | | | **Reference** | |
| --- | --- | --- | --- | --- | --- | --- | --- |
| **pGEM**®**-T easy** | | | Cloning vector, Ap^r^ | | | Promega | |
| **pBlueScript II KS+** | | | Cloning vector, Ap^r^ | | | Stratagene | |
| **pMP220** | | | Promoter probe vector, IncP, LacZ, Tc^r^ | | | ([Spaink *et al.*, 1987](#_ENREF_9)) | |
| **pMP190** | | | Promoter probe vector, IncW, LacZ, Cm^r^ | | | ([Spaink *et al.*, 1987](#_ENREF_9)) | |
| **pSS122** | | | Promoter probe vector, IncW, Ap^r^, Gm^r^ | | | ([Ferluga and Venturi, 2009](#_ENREF_3)) | |
| **pBBR1MCS2** | | | Broad-host-range vector, Km^r^ | | | ([Kovach *et al.*, 1995](#_ENREF_6)) | |
| **pBBR1MCS3** | | | Broad-host-range vector, Tc^r^ | | | ([Kovach *et al.*, 1995](#_ENREF_6)) | |
| **pBBR1MCS5** | | | Broad-host-range vector, Gm^r^ | | | ([Kovach *et al.*, 1995](#_ENREF_6)) | |
| **pUC57** | | | Cloning vector, Ap^r^ | | | Genscript | |
| **pMX** | | | Cloning vector, Ap^r^ | | | ([Kitamura *et al.*, 2003](#_ENREF_5)) | |
| **pLASI220** | | | *lasI* promoter of *P. aeruginosa* cloned in pMP220, Tc^r^ | | | ([Babic *et al.*, 2010](#_ENREF_1)) | |
| **pLASI190** | | | *lasI* promoter of *P. aeruginosa* cloned in pMP190, Cm^r^ | | | ([Rampioni *et al.*, 2007](#_ENREF_8)) | |
| **pPPUI220** | | | *ppuI* promoter of *P. putida* cloned in pMP220, Tc^r^ | | | ([Bertani and Venturi, 2004](#_ENREF_2)) | |
| **pCVII220** | | | *cviI* promoter of *C. violaceum* cloned in pMP220, Tc^r^ | | | ([Suarez-Moreno *et al.*, 2010](#_ENREF_11)) | |
| **pGLUI220** | | | *gluI* promoter of *B. glumae* cloned in pMP220, Tc^r^ | | | ([Suarez-Moreno *et al.*, 2010](#_ENREF_47)) | |
| **pPIP220** | | | *pip* promoter of *X. oryzae* cloned in pMP220, Tc^r^ | | | ([Subramoni *et al.*, 2011](#_ENREF_12)) | |
| **pPIP122** | | *pip* promoter of *X. oryzae* cloned in pSS122, Ap^r^, Gm^r^ | | | ([Ferluga and Venturi, 2009](#_ENREF_3)) | |  |
| **Primer** | **Primer sequence** | | | **Amplicon/insert size (bp)** | | |  |
| **LasR Fw**  **LasR Rv** | | | AAGCTTCGAATCCATATTTGGCTG  GAGCTCACCTGAGAGGCAAGAT | | | 720 |  |
| **RhlR Fw**  **RhlR Rv** | | | AAGCTTATCGATCAGGGCTTACT  GAGCTCTGCGCTTCAGATGAGA | | | 726 |  |
| **T3**  **RhlRSmaI Rv** | | | ATTAACCCTCACTAAAGGGA  CAGCCCGGGTTGGACAT | | | 520 |  |

Ap^r^, Km^r^, Tc^r^, Gm^r^ and Cm^r^, resistant to ampicillin, kanamycin, tetracycline, gentamicin and chloramphenicol, respectively.

**Table S3.** List of synthetic constructs created in this study.

| **Plasmids** | **Sequence** |
| --- | --- |
| **pRHLAS** | ATGAGGAATGACGGAGGCTTTTTGCTGTGGTGGGACGGTTTGCGTAGCGAGATGCAGCCGATCCACGACAGCCAGGGCGTGTTCGCCGTCCTGGAAAAGGAAGTGCGGCGCCTGGGCTTCGATTACTACGCCTATGGCGTGCGCCATACGATTCCCTTCACCCGGCCGAAGACCGAGGTCCATGGCACCTATCCCAAGGCCTGGCTGGAGCGATACCAGATGCAGAACTACGGGGCCGTGGATCCGGCGATCCTCAACGGCCTGCGCTCCTCGGAAATGGTGGTCTGGAGCGACAGCCTGTTCGACCAGAGCCGGATGCTCTGGAACGAGGCTCGCGATTGGGGCCTCTGTGTCGGCGCGACCCTGCCGATCCGCGCGCCGAACAATTTGCTCAGCGTGCTTTCCGTGGCGCGCGACCAGCAGAACATCTCCAGCTTCGAGCGCGAGGAAATACGCCTGCGGCTGCGTTGCATGATCGAGTTGCTGACCCAGAAGCTGACCGACCTGGAGCATCCGATGCTGATGTCCAACCCCGGTGCCGGACTGGCCTTCGAACATCCGGTCAGCAAACCGGTGGTTCTGACCAGCCGGGAGAAGGAAGTGTTGCAGTGGTGCGCCATCGGCAAGACCAGTTGGGAGATATCGGTTATCTGCAACTGCTCGGAAGCCAATGTGAACTTCCATATGGGAAATATTCGGCGGAAGTTCGGTGTGACCTCCCGCCGCGTAGCGGCCATTATGGCCGTTAATTTGGGTCTTATTACTCTCTGA |
| **pLAO** | AAGCTTAAAGAGGAAGAAATACTATGGCCTTGGTTGACGGTTTTCTTGAGCTGGAACGCTCAAGTGGAAAATTGGAGTGGAGCGCCATCCTGCAGAAGATGGCGAGCGACCTTGGATTCTCGAAGATCCTGTTCGGCCTGTTGCCTAAGGACAGCCAGGACTACGAGAACGCCTTCATCGTCGGCAACTACCCGGCCGCCTGGCGCGAGCATTACGACCGGGCTGGCTACGCGCGGGTCGACCCGACGGTCAGTCACTGTACCCAGAGCGTACTGCCGATTTTCTGGGAACCGTCCATCTACCAGACGCGAAAGCAGCACGAGTTCTTCGAGGAAGCCTCGGCCGCCGGCCTGGTGTATGGGCTGACCATGCCGCTGCATGGTGCTCGCGGCGAACTCGGCGCGCTGAGCCTCAGCGTGGAAGCGGAAAACCGGGCCGAGGCCAACCGTTTCATGGAGTCGGTCCTGCCGACCCTGTGGATGCTCAAGGACTACGCACTGCAGAGCGGTGCCGGACTGGCCTTCGAACATCCGGTCAGCAAACCGCCATTGACCCGTCGCGAGCGCGAATGCCTGCAGTATTCGGCCAAAGGCCTGACCTCCAAACGTATCGCCGCGGCGCTCAACCGCTCCACCGCCACGGTGAACCTGCATCTGAATTCGGCTGCCCGCAAACTGGGGGCACGTAACCGCGTGGAAGCGGTGGTGCGTGGTATGCACTATCGGTTGCTGGAGCCATAAGAGCTC |
| **pOLA** | TAAAGCTTAAAGAGGAAGAAATACTATGTTCGAAATTCTAGCCAGCCTGGGCCGCGATCTGCAGGCGTCGCAAACGGTTAATAGCTGCCTGGATCGGGTGTTTCGCGATGTCTGTGCGCTCGGCTTCCAGTCGTTGGTCTACGACTACGCACCGGTGCCGCTGAGCATGGAGGGCGCGCTGATCACGCCAACGGTGTTCATGCAGCGCAATGCGCCAGGCGATATGCAGCATGTCTGGTGCGAGCACGGGTACTACCAACATGACCCCGTTCAGCAGCGTGCAACGCGACGTAACACCCCGTTCGTATGGTCGTACCGCACCGACGGCGATTGCGCTGGGGTGGAATATGTGGGTGGACAGCACCGGCAAGTCACGCGTTACTTGTGCGATAGCGGCATGGGTACCGGTGTCACCGTGCCGCTGCATCTGCCCGGTGGCGCGTTCGCCACCTTTAGCGCTGCGATTGATGCCGTGGCTGCGGAAGCGCTGCGTCTGGCCGAGTCGCAGTTATTGCCCTTCTTGCTGCTGGCACATGCTTTCCAGGCGCGTGCGCAGGAATTGCTGGACCCGCAGGAACGCCGCTGCCACCACATTCCGGTGGTTCTGACCAGCCGGGAGAAGGAAGTGTTGCAGTGGTGCGCCATCGGCAAGACCAGTTGGGAGATATCGGTTATCTGCAACTGCTCGGAAGCCAATGTGAACTTCCATATGGGAAATATTCGGCGGAAGTTCGGTGTGACCTCCCGCCGCGTAGCGGCCATTATGGCCGTTAATTTGGGTCTTATTACTCTCTGAGAGCTCAT |
| **pDAHL1** | AAGCTTAAAGAGGAAGAAATACTATGAGGAATGACGGAGGCTTTTTGCTGTGGTGGGACGGTTTGCGTAGCGAGATGCAGCCGATCCACGACAGCCAGGGCGTGTTCGCCGTCCTGGAAAAGGAAGTGCGGCGCCTGGGCTTCGATTACTACGCCTATGGCGTGCGCCACACGATTCCCTTCACCCGGCCGAAGACCGAGGTCCATGGCACCTATCCCAAGGCCTGGCTGGAGCGATACCAGATGCAGAACTACGGGGCCGTGGATCCGGCGATCCTCAACGGCCTGCGCTCCTCGGAAATGGTGGTCTGGAGCGACAGCCTGTTCGACCAGAGCCGGATGCTCTGGAACGAGGCTCGCGATTGGGGCCTCTGTGTCGGCGCGACCTTGCCGATCCGCGCGCCGAACAATTTGCTCAGCGTGCTTTCCGTGGCGCGCGACCAGCAGAACATCTCCAGCTTCGAGCGCGAGGAAATCCGCCTGCGGCTGCGTTGCATGATCGAGTTGCTGACCCAGAAGCTGACCGACCTGGAGCATCCGATGCTGATGTCCAACCCGCTCAGAATGGCCTTGGTTGACGGTTTTCTTGAGCTGGAACGCTCAAGTGGAAAATTGGAGTGGAGCGCCATCCTGCAGAAGATGGCGAGCGACCTTGGATTCTCGAAGATCCTGTTCGGCCTGTTGCCTAAGGACAGCCAGGACTACGAGAACGCCTTCATCGTCGGCAACTACCCGGCCGCCTGGCGCGAGCATTACGACCGGGCTGGCTACGCGCGGGTCGACCCGACGGTCAGTCACTGTACCCAGAGCGTACTGCCGATTTTCTGGGAACCGTCCATCTACCAGACGCGAAAGCAGCACGAGTTCTTCGAGGAAGCCTCGGCCGCCGGCCTGGTGTATGGGCTGACCATGCCGCTGCATGGTGCTCGCGGCGAACTCGGCGCGCTGAGCCTCAGCGTGGAAGCGGAAAACCGGGCCGAGGCCAACCGTTTCATGGAGTCGGTCCTGCCGACCCTGTGGATGCTCAAGGACTACGCACTGCAGAGCGGTGCCGGACTGGCCTTCGAACATCCGGTCAGCAAACCGGTGGTTCTGACCAGCCGGGAGAAGGAAGTGTTGCAGTGGTGCGCCATCGGCAAGACCAGTTGGGAGATATCGGTTATCTGCAACTGCTCGGAAGCCAATGTGAACTTCCATATGGGAAATATTCGGCGGAAGTTCGGTGTGACCTCCCGCCGCGTAGCGGCCATTATGGCCGTTAATTTGGGTCTTATTACTCTCTGAGAGCTC |
| **pDAHL2** | GAATTCAAAGAGGAAGAAATACTATGGCCTTGGTTGACGGTTTTCTTGAGCTGGAACGCTCAAGTGGAAAATTGGAGTGGAGCGCCATCCTGCAGAAGATGGCGAGCGACCTTGGATTCTCGAAGATCCTGTTCGGCCTGTTGCCTAAGGACAGCCAGGACTACGAGAACGCCTTCATCGTCGGCAACTACCCGGCCGCCTGGCGCGAGCATTACGACCGGGCTGGCTACGCGCGGGTCGACCCGACGGTCAGTCACTGTACCCAGAGCGTACTGCCGATTTTCTGGGAACCGTCCATCTACCAGACGCGAAAGCAGCACGAGTTCTTCGAGGAAGCCTCGGCCGCCGGCCTGGTGTATGGGCTGACCATGCCGCTGCATGGTGCTCGCGGCGAACTCGGCGCGCTGAGCCTCAGCGTGGAAGCGGAAAACCGGGCCGAGGCCAACCGTTTCATGGAGTCGGTCCTGCCGACCCTGTGGATGCTCAAGGACTACGCACTGCAGAGCGGTGCCGGACTGGCCTTCGAACATCCGGTCAGCAAAATGAGGAATGACGGAGGCTTTTTGCTGTGGTGGGACGGTTTGCGTAGCGAGATGCAGCCGATCCACGACAGCCAGGGCGTGTTCGCCGTCCTGGAAAAGGAAGTGCGGCGCCTGGGCTTCGATTACTACGCCTATGGCGTGCGCCACACGATTCCCTTCACCCGGCCGAAGACCGAGGTCCATGGCACCTATCCCAAGGCCTGGCTGGAGCGATACCAGATGCAGAACTACGGGGCCGTGGATCCGGCGATCCTCAACGGCCTGCGCTCCTCGGAAATGGTGGTCTGGAGCGACAGCCTGTTCGACCAGAGCCGGATGCTCTGGAACGAGGCTCGCGATTGGGGCCTCTGTGTCGGCGCGACCTTGCCGATCCGCGCGCCGAACAATTTGCTCAGCGTGCTTTCCGTGGCGCGCGACCAGCAGAACATCTCCAGCTTCGAGCGCGAGGAAATCCGCCTGCGGCTGCGTTGCATGATCGAGTTGCTGACCCAGAAGCTGACCGACCTGGAGCATCCGATGCTGATGTCCAACCCGCCGGTGGTTCTGACCAGCCGGGAGAAGGAAGTGTTGCAGTGGTGCGCCATCGGCAAGACCAGTTGGGAGATATCGGTTATCTGCAACTGCTCGGAAGCCAATGTGAACTTCCATATGGGAAATATTCGGCGGAAGTTCGGTGTGACCTCCCGCCGCGTAGCGGCCATTATGGCCGTTAATTTGGGTCTTATTACTCTCTGATCTAGA |

**REFERENCES**

Babic, F., Venturi, V., and Maravic-Vlahovicek, G. (2010). Tobramycin at subinhibitory concentration inhibits the RhlI/R quorum sensing system in a Pseudomonas aeruginosa environmental isolate. *BMC Infect Dis* 10**,** 148.

Bertani, I., and Venturi, V. (2004). Regulation of the N-acyl homoserine lactone-dependent quorum-sensing system in rhizosphere Pseudomonas putida WCS358 and cross-talk with the stationary-phase RpoS sigma factor and the global regulator GacA. *Appl Environ Microbiol* 70**,** 5493-5502.

Ferluga, S., and Venturi, V. (2009). OryR is a LuxR-family protein involved in interkingdom signaling between pathogenic *Xanthomonas oryzae* pv. *oryzae* and rice. *J Bacteriol* 191**,** 890-897.

Figurski, D.H., and Helinski, D.R. (1979). Replication of an origin-containing derivative of plasmid RK2 dependent on a plasmid function provided in trans. *Proc Natl Acad Sci U S A* 76**,** 1648-1652.

Kitamura, T., Koshino, Y., Shibata, F., Oki, T., Nakajima, H., Nosaka, T., and Kumagai, H. (2003). Retrovirus-mediated gene transfer and expression cloning: powerful tools in functional genomics. *Exp Hematol* 31**,** 1007-1014.

Kovach, M.E., Elzer, P.H., Hill, D.S., Robertson, G.T., Farris, M.A., Roop, R.M., 2nd, and Peterson, K.M. (1995). Four new derivatives of the broad-host-range cloning vector pBBR1MCS, carrying different antibiotic-resistance cassettes. *Gene* 166**,** 175-176.

Kumar, R.S., Ayyadurai, N., Pandiaraja, P., Reddy, A.V., Venkateswarlu, Y., Prakash, O., and Sakthivel, N. (2005). Characterization of antifungal metabolite produced by a new strain Pseudomonas aeruginosa PUPa3 that exhibits broad-spectrum antifungal activity and biofertilizing traits. *J Appl Microbiol* 98**,** 145-154.

Rampioni, G., Schuster, M., Greenberg, E.P., Bertani, I., Grasso, M., Venturi, V., Zennaro, E., and Leoni, L. (2007). RsaL provides quorum sensing homeostasis and functions as a global regulator of gene expression in Pseudomonas aeruginosa. *Mol Microbiol* 66**,** 1557-1565.

Spaink, H.P., Okker, R.J., Wijffelman, C.A., Pees, E., and Lugtenberg, B.J. (1987). Promoters in the nodulation region of the *Rhizobium leguminosarum* Sym plasmid pRL1JI. *Plant Mol Biol* 9**,** 27-39.

Steindler, L., Bertani, I., De Sordi, L., Schwager, S., Eberl, L., and Venturi, V. (2009). LasI/R and RhlI/R quorum sensing in a strain of *Pseudomonas aeruginosa* beneficial to plants. *Appl Environ Microbiol* 75**,** 5131-5140.

Suarez-Moreno, Z.R., Devescovi, G., Myers, M., Hallack, L., Mendonca-Previato, L., Caballero-Mellado, J., and Venturi, V. (2010). Commonalities and differences in regulation of N-acyl homoserine lactone quorum sensing in the beneficial plant-associated burkholderia species cluster. *Appl Environ Microbiol* 76**,** 4302-4317.

Subramoni, S., Gonzalez, J.F., Johnson, A., Pechy-Tarr, M., Rochat, L., Paulsen, I., Loper, J.E., Keel, C., and Venturi, V. (2011). Bacterial subfamily of LuxR regulators that respond to plant compounds. *Appl Environ Microbiol* 77**,** 4579-4588.
