# Supplementary material for: Studies on synthetic LuxR solo hybrids
Source: Front Cell Infect Microbiol. 2015 Jun 18;5:52. doi: 10.3389/fcimb.2015.00052 (PMC4471428; doi:10.3389/fcimb.2015.00052)
Supplement: Supplementary file 2 [file Image1.PDF]

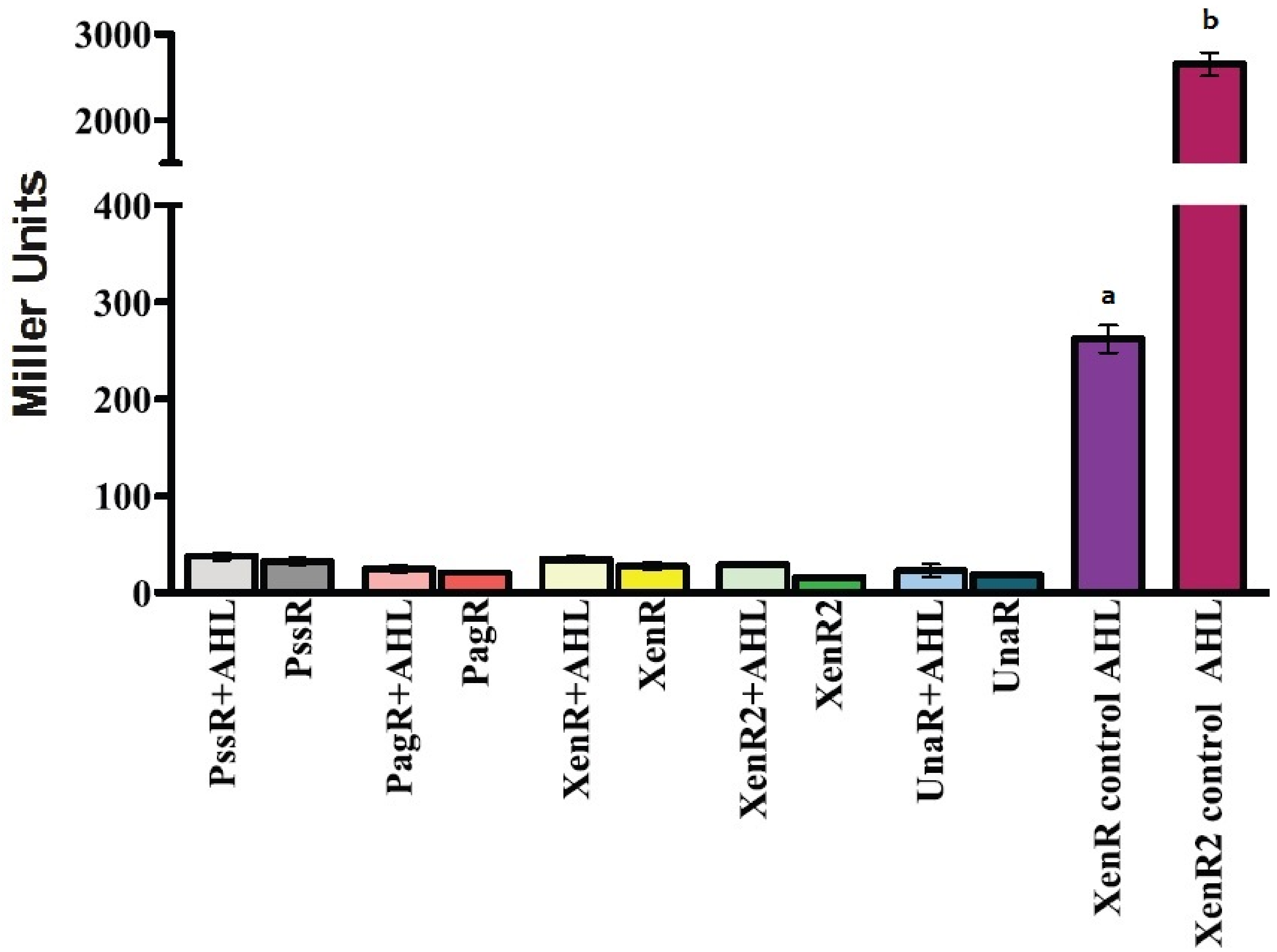

Figure S1. β-galactosidase assays showing reporter expression levels regulated by *pip* and *xenI* promoters in *E. coli*. (A) β-galactosidase measurements of promoter activities in the presence of several native luxR homologs (PssR, PagR, XenR, XenR2 and UnaR) using plasmids pPIP220 and pXENI220 in the absence or presence of AHLs. All experiments were performed on triplicate, the means and errors are shown and the statistical analysis were calculated using Student's t-test (P≤0.05). Distinct letters (a and b) indicate statistically different values.
