# Supplementary material for: Studies on synthetic LuxR solo hybrids
Source: Front Cell Infect Microbiol. 2015 Jun 18;5:52. doi: 10.3389/fcimb.2015.00052 (PMC4471428; doi:10.3389/fcimb.2015.00052)
Supplement: Supplementary file 3 [file Image2.PDF]

Supplementary Material

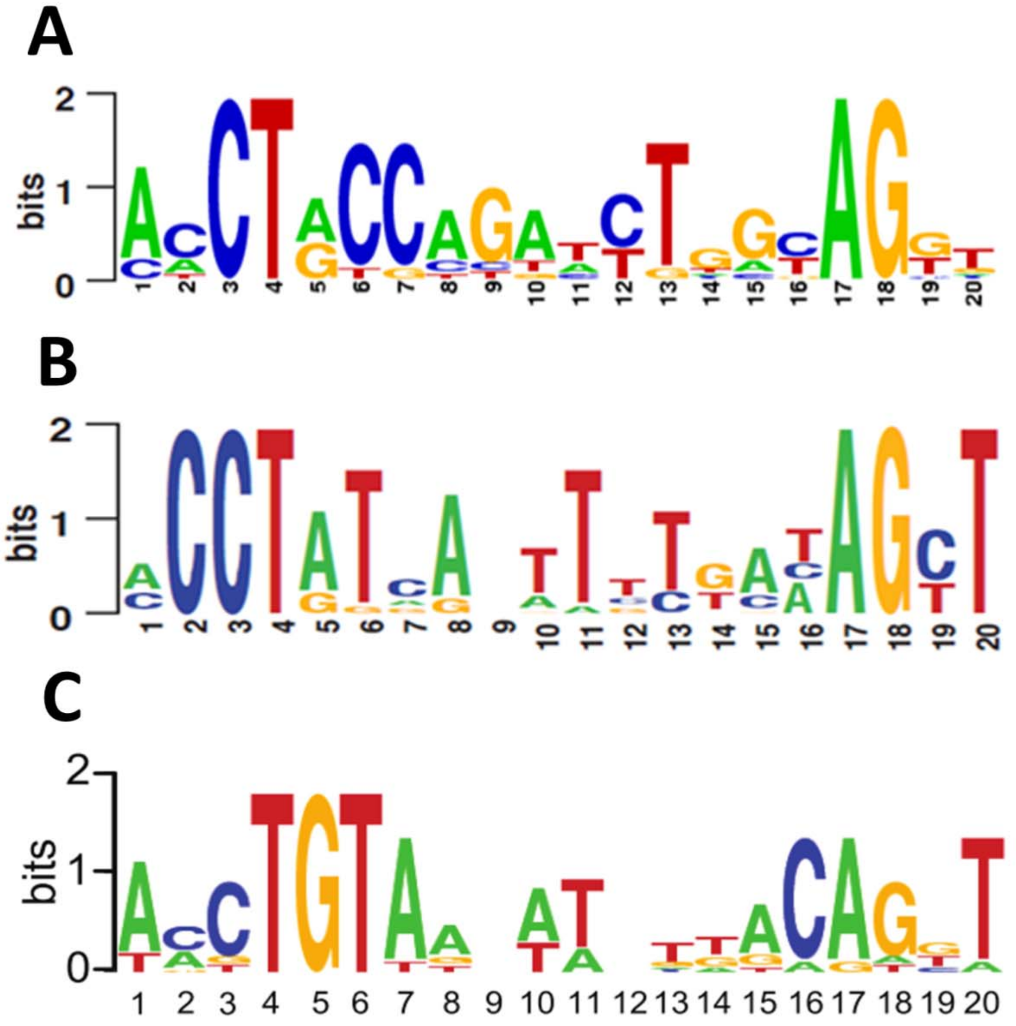

Figure S2. *lux* box prediction of *luxR, lasR* and *oryR* target regions. Predicted *lux*-like boxes found in the promoters activated by (A) LasR, (B) OryR and LuxR (C) using Weblogo (http://weblogo.berkeley.edu/). Presence of conserved CT(N_{12})AG motif on (A) and (B) boxes.
