# Supplementary material for: Studies on synthetic LuxR solo hybrids
Source: Front Cell Infect Microbiol. 2015 Jun 18;5:52. doi: 10.3389/fcimb.2015.00052 (PMC4471428; doi:10.3389/fcimb.2015.00052)
Supplement: Supplementary file 4 [file Image3.PDF]

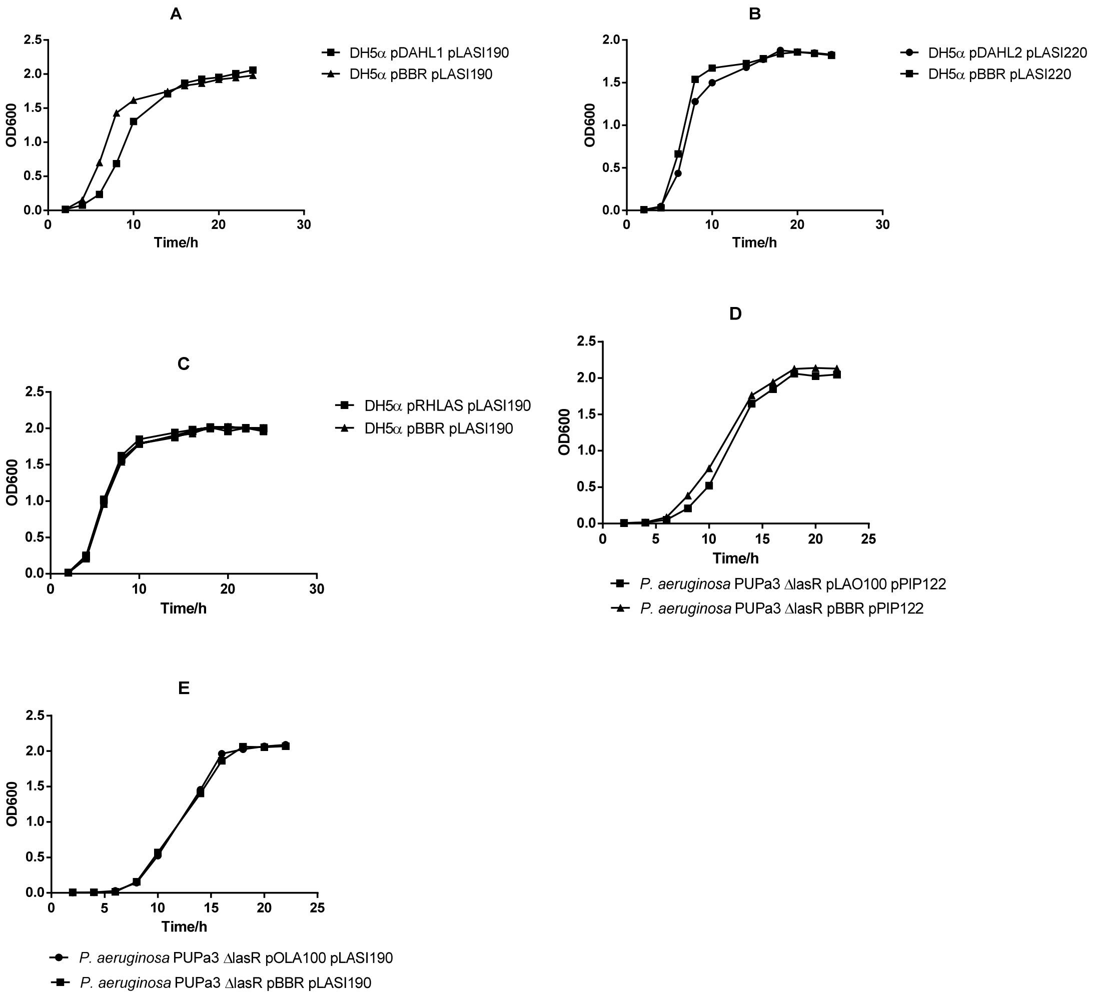

Figure S3. **Growth curves of strains used in this study harboring either empty vectors or hybrid constructs.** Growth curve of DH5α pDAHL1 pLASI190 (A), DH5α pDAHL2 pLASI220 (B), DH5α pRHLAS pLASI190 (C), *P. aeruginosa* PUPa3 Δ*lasR* pLAO100 pPIP122 (D) and *P. aeruginosa* PUPa3 Δ*lasR* pOLA100 pLASI190 (E). All experiments have the pBBR empty plasmid as control. All experiments were performed in triplicate resulting in similar results..
